# Urinary Exosomes Diagnosis of Urological Tumors: A Systematic Review and Meta-Analysis

**DOI:** 10.3389/fonc.2021.734587

**Published:** 2021-09-10

**Authors:** Yipeng Xu, Jianmin Lou, Mingke Yu, Yingjun Jiang, Han Xu, Yueyu Huang, Yun Gao, Hua Wang, Guorong Li, Zongping Wang, An Zhao

**Affiliations:** ^1^Department of Urology, Cancer Hospital of University of Chinese Academy of Sciences, Zhejiang Cancer Hospital, Hangzhou, China; ^2^Zhejiang Chinese Medical University, Hangzhou, China; ^3^Hangzhou Traditional Chinese Medicine Hospital, Zhejiang Chinese Medical University, Hangzhou, China; ^4^Central Research Laboratory, Children’s Hospital of Nanchang University, Nanchang, China; ^5^Experimental Research Center, Cancer Hospital of University of Chinese Academy of Sciences, Zhejiang Cancer Hospital, Hangzhou, China; ^6^Institute of Cancer and Basic Medicine (ICBM), Chinese Academy of Sciences, Hangzhou, China; ^7^Department of Urology, North Hospital, CHU of Saint-Etienne, University of Jean-Monnet, Saint-Etienne, France; ^8^Inserm U1059, Faculty of Medicine, University of Jean-Monnet, Saint-Etienne, France

**Keywords:** urological tumor, exosomes, urine, diagnosis, liquid biopsy

## Abstract

**Purpose:**

Exosomes could be released directly into the urine by the urological tumoral cells, so testing urinary exosomes has great potential for non-invasive diagnosis and monitor of urological tumors. The objective of this study is to systematically review and meta-analysis of urinary exosome for urological tumors diagnosis.

**Materials and Methods:**

A systematic review of the recent English-language literature was conducted according to the PRISMA statement recommendations (CRD42021250613) using PubMed, Embase, Cochrane Library, Web of Science, and Scopus databases up to April 30, 2021. Risk-of-bias assessment was performed according to the QUADAS 2 tool. The true diagnostic value of urinary exosomes by calculating the number of true positive, false positive, true negative, and false negative, diagnoses by extracting specificity and sensitivity data from the selected literature.

**Results:**

Sixteen eligible studies enrolling 3224 patients were identified. The pooled sensitivity and specificity of urinary exosomes as a diagnostic tool in urological tumors were 83% and 88%, respectively. The area under the summary receiver operating characteristic curve was 0.92 (95% CI: 0.89–0.94). Further subgroup analyses showed that our results were stable irrespective of the urinary exosome content type and tumor type.

**Conclusion:**

Urinary exosomes may serve as novel non-invasive biomarkers for urological cancer detection. Future clinical trial designs must validate and explore their utility in treatment decision-making.

**Systematic Review Registration:**

[ https://www.crd.york.ac.uk/prospero/], identifier [CRD42021250613].

## Introduction

Tissue biopsy is the current standard method for pathological diagnosis of urological cancer. However, based on one single needle biopsy is limited in reflecting the complete genomic landscape of cancer accurately and is inappropriate for early tumor screening (1). To detect cell-free biomarkers (such as circulating nucleic acids, circulating tumor cells and circulating exosomes) in the body fluid, also called “Liquid biopsy”, has recently show its value in clinical application (2). Collecting the circulating tumor related gene has the potential to provide molecular characterization of primary or metastatic tumor, and these cell-free biomarkers may be used to manage the post-treatment process of tumor (3).

One of the main types of liquid biopsies, circulating exosome, is extracellular vesicles enclosed by a lipid bilayer membrane range from 40 to 150 nm. Exosomes contain a complex cargo of contents derived from the original cell, including nucleic acids, lipids, and proteins (4). The exosome released by tumor cells has been shown to play an important role in microenvironment, immune regulation, and other malignant processes (5). Compared with other tumors, urological tumors can direct release exosomes into the urine, so urinary exosomes may be more sensitive and specific to reflect the status of urological tumors (6). Since then, several studies assessing the diagnostic value of urinary exosome in urological tumor have been published (5, 7). But the diagnostic performance of this novel biomarker has not been evaluated systematically. Therefore, the purpose of this study was to assess the diagnostic performance of urinary exosome for the detection of urological cancer including renal cancer (RCa), bladder cancer (BCa), and prostate cancer (PCa).

## Materials and Methods

The protocol has been registered in the International Prospective Register of Systematic Reviews database (registration number: CRD42021250613).

### Search Strategy

This systematic review and meta-analysis were performed according to the Preferred Reporting Items for Systematic Reviews and Meta-analyses (PRISMA) statement (8). A comprehensive literature search was followed the PRISMA 2009 checklist, and the PubMed, Embase, Cochrane Library, Web of Science, and Scopus databases were searched systematically in April 30, 2021.

The search strategy included the following terms: (“exosomes” or “extracellular vesicle”) AND “urine” AND (“diagnosis” OR “biomarker”) AND (“urological cancer” OR “urologic neoplasms” OR “urogenital neoplasms”) AND (“kidney neoplasms” OR “kidney cancer” OR “renal cancer”) AND (“prostate neoplasms” OR “prostate cancer”) AND (“bladder cancer” OR “bladder neoplasms”). Two researchers (Yipeng Xu and Jianmin Lou) independently assessed the eligibility of each potentially relevant study by screening the titles and abstracts. Disagreements between the two researchers were resolved by discussion with two additional researchers (An Zhao and Zongping Wang). Other publications were identified by searching the list of references of the selected papers.

### Inclusion and Exclusion Criteria

Inclusion criteria for primary studies were as follows: (1) The research article was a diagnostic study using urinary exosomes; (2) Subjects included cancer patients and healthy controls; (3) The data was sufficient to generate a two-by-two table consisting of true negative (TN), and false negative (FN), true positive (TP), and false positive (FP).

The exclusion criteria were as follows: (1) repeated or overlapped publications which included the same study population and genes; (2) experiments based exclusively on cell lines or tumor tissue rather than clinical samples; and (3) studies with a poor sample size (≤10).

### Data Extraction and Quality Assessment

We extracted the following data from the selected studies: the first author’s last name, year of publication, country of study, cancer type, sample sizes, exosome extraction method, type of exosome content/detection method, target molecular detection, diagnostic results (numbers of FP, FN, TP, and TN), and diagnostic performance (sensitivity and specificity).

Deek’s funnel plot and Quality Assessment of Diagnostic Accuracy Studies (QUADAS) 2 tool were adopted to analyze qualitative publication bias, and a P-value of <0.05 was considered statistically significant. Risk-of-bias assessment was performed independently by two authors (YJ, YX) according to the QUADAS 2 tool. Disagreement was solved by a third party (AZ). This tool provides a measure of the risk of bias and applicability over four domains (index test, reference standard, flow, and timing) of interest (9).

### Data Synthesis and Analysis

All statistical analyses were performed using STATA software (version 12.0, STATA Corp, MIDAS module). Quality assessment was managed with Review Manager 5.3 (Cochrane Collaboration, Copenhagen, Denmark). The number of diagnoses (TP, TN, FP, and FN) from each study was extracted to calculate diagnostic sensitivity, specificity, positive likelihood ratio (PLR), negative likelihood ratio (NLR), and diagnostic odds ratio (DOR) with 95% confidence interval (CIs). PLR is calculated as sensitivity/(1-specifcity), and NLR is calculated as (1-sensitivity)/specificity. The DOR value is used as a measure of the effectiveness of a diagnostic test and is calculated as PLR/NLR. Summary ROC curves (SROC) and AUCs of the SROC were measured. All P values were two sided, and a P value < 0.05 was considered as statistically significant.

## Results

### Literature Search

**Figure 1:**
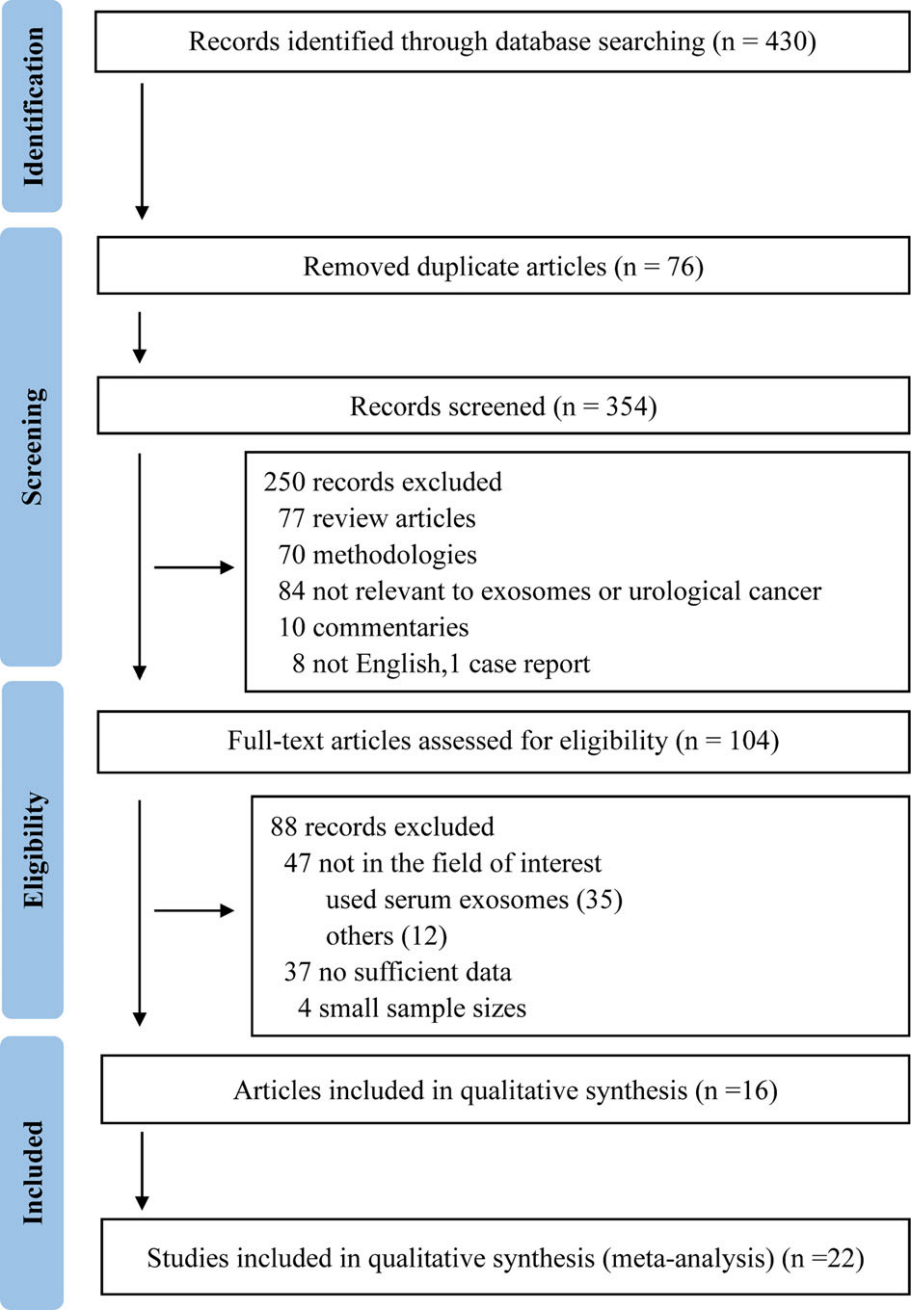
PRISMA flow diagram showing study selection process for meta-analysis.

Four hundred and thirty studies were confirmed through systematic search and manual review for initial screening, and 354 studies were remained after duplicates removed. After titles and abstracts were checked, 104 articles of the non-duplicate records were subjected to further full-text review, of which 88 were excluded according to the exclusion criteria. Finally, 22 studies from 16 articles were included in the present meta-analysis (10–25). No additional studies were identified *via* screening the bibliographies of these 16 articles. The process of literature inclusion and selection is presented in **Figure 1**.

### Characteristics of Included Studies

Among them, 5 eligible studies featured a total of 408 patients with bladder cancer, 9 eligible studies featured a total of 1277 patients with prostate cancer, and 2 eligible studies featured a total of 179 patients with renal cell carcinoma. The main extraction methods of urinary exosome are ultracentrifugation or commercial exosome extraction kit. The technique for molecular examination depends on the type of exosome contents, nucleic acid exosome cargo was detected using methods such as qRT-PCR or sequencing, and non-nucleic acid exosomal cargo (proteins or lipids) was detected using methods such as enzyme-linked immunosorbent assay (ELISA) or mass spectrometry (MS). In total, all main characteristics of the eligible studies were summarized (**Table 1**).

**Table 1:** Characteristics of studies evaluating the urinary exosomes of patients with urological tumor.

Study ID (Ref/Region)	Sample size (case/control)	Exosome extraction method	Type of exosome content/detection method	Target molecular detection	TP	FP	TN	FN	Sensitivity	Specificity
***Bladder cancer***										
10 **/China**	104/104 (Training set)	Urine Exosome RNA Isolation Kit (Norgen Biotek, Thorold, Canada)	Nucleic acid/ qRT-PCR	Panel of lncRNAs (MALAT1+PCAT-1+SPRY4-IT1)	75	19	89	29	72.1%	85.6%
	80/80 (Validation set)				50	12	68	30	62.5%	85.0%
11 **/Iran**	59/24	Urine Exosome RNA Isolation Kit (Norgen Biotek, Thorold, Canada)	Nucleic acid/ qRT-PCR	Panel of lncRNAs (UCA1-201+UCA1-203+ MALAT1+LINC00355)	54	2	22	5	91.5%	91.7%
12 **/Turkey**	59/34	Urine Exosome RNA Isolation Kit (Norgen Biotek, Thorold, Canada)	Nucleic acid/ qRT-PCR	Panel of miRNAs (miR-19b1-5p+miR-136-3p+ miR139-5p)	52	7	27	7	80.0%	88.1%
13 **/Egypt**	70/12	Centrifugation, Filtration	Non-Nucleic acid/ Elisa	CD9 protein	65	2	10	5	92.6%	83.3%
14 **/Japan**	36/24	Ultracentrifugation	Nucleic acid/ qRT-PCR/	miR-21-5p	27	1	23	9	75.0%	95.8%
***Renal cell carcinoma***										
15 **/China**	70/30	Ultracentrifugation	Nucleic acid/ qRT-PCR	miR-30c-5p	48	0	30	22	68.6%	100.0%
16 **/Canada**	28/18 (Discovery set)	Urine Exosome RNA Isolation Kit (Norgen Biotek, Thorold, Canada)	Nucleic acid/ qRT-PCR	Panel of miRNAs (miR-126-3p+miR-449a, the best combination)	23	5	13	5	82.8%	70.0%
	81/33 (Validation set)				68	12	21	13	83.8%	62.5%
***Prostate cancer***										
17 **/USA**	568/268 (Training set)	Exosome RNA Isolation Kits (Norgen Biotek, Ontario, Canada)	Nucleic acid/ QuantStudio OpenArray	Panel of sncRNAs (Selected miRNAs+ selected snoRNAs)	533	11	257	35	93.8%	95.9%
	300/300 (Validation set)				281	25	275	19	93.7%	91.7%
18 **/Norway**	20/9	Sequential centrifugation	Nucleic acid/ NGS	miR-196a	20	1	8	0	100.0%	88.9%
19 **/Russia**	14/20 (TEV set)	Ultracentrifugation	Nucleic acid/ qRT-PCR	miR-19b	13	0	20	1	92.9%	100.0%
	14/20 (ERV set)				11	1	19	3	78.6%	95.0%
20 **/USA**	89/106	Urine exosome clinical sample concentrator kit (Exosome Diagnostics, Cambridge, MA, USA)	Nucleic acid/ qRT-PCR	Panel of mRNAs (PCA3 and ERG)	67	49	57	22	75.3%	53.8%
21 **/Canada**	28/28	Sucrose cushion ultracentrifugation	Nucleic acid/ qRT-PCR	Panel of mRNAs and miRNAs (ANXA3, CD24, TMPRSS2-ERG, SLC45A3, FOLH1, HPN, ITSN1, miR-375-3p, miR-574-3p)	22	3	25	6	78.6%	89.3%
22 **/Netherlands**	48/26	Ultracentrifugation	Nucleic acid/ qRT-PCR	Panel of miRNA isoforms (isomiRs of miR−21, miR−204 and miR−375)	35	3	23	13	72.9%	88.5%
23 **/Belgium**	85/122 (Overall population set)	N-butanol (Sigma-Aldrich, St. Louis, Missouri, USA), Ultracentrifugation	Non-Nucleic acid/ ECLIA	Urinary vesicle-associated PSA extraction ratio	60	55	67	25	70.6%	54.9%
	61/56 (sPSA between 4 ug/L and 10 ug/L set)				39	22	34	22	63.9%	60.7%
24 **/Norway**	15/15	Sequential centrifugation	Non-Nucleic acid/ MS	Panel of lipids (LacCer; d18:1/16:0, PS; 18:1/18:1 and 18:0/18:2)	14	0	15	1	93.3%	100%
25 **/Norway**	16/16	Sequential centrifugation	Non-Nucleic acid/ WB, ELISA	Flotillin 2 protein	14	1	15	2	87.5%	93.8%
	19/15			Panel of proteins (Flotillin 2 and PARK7)	13	1	14	6	68.4%	93.3%

TP, true positive; FP, false positive; TN, true negative; FN, false negative; qRT-PCR, quantitative real-time polymerase chain reaction; ELISA, enzyme-linked immunosorbent assay; NGS, next generation sequencing; MS, mass spectrometry; WB, Western blotting; ECLIA, electrochemiluminescence immunoassay.

### Risk of Bias Within Studies

**Figure 2:**
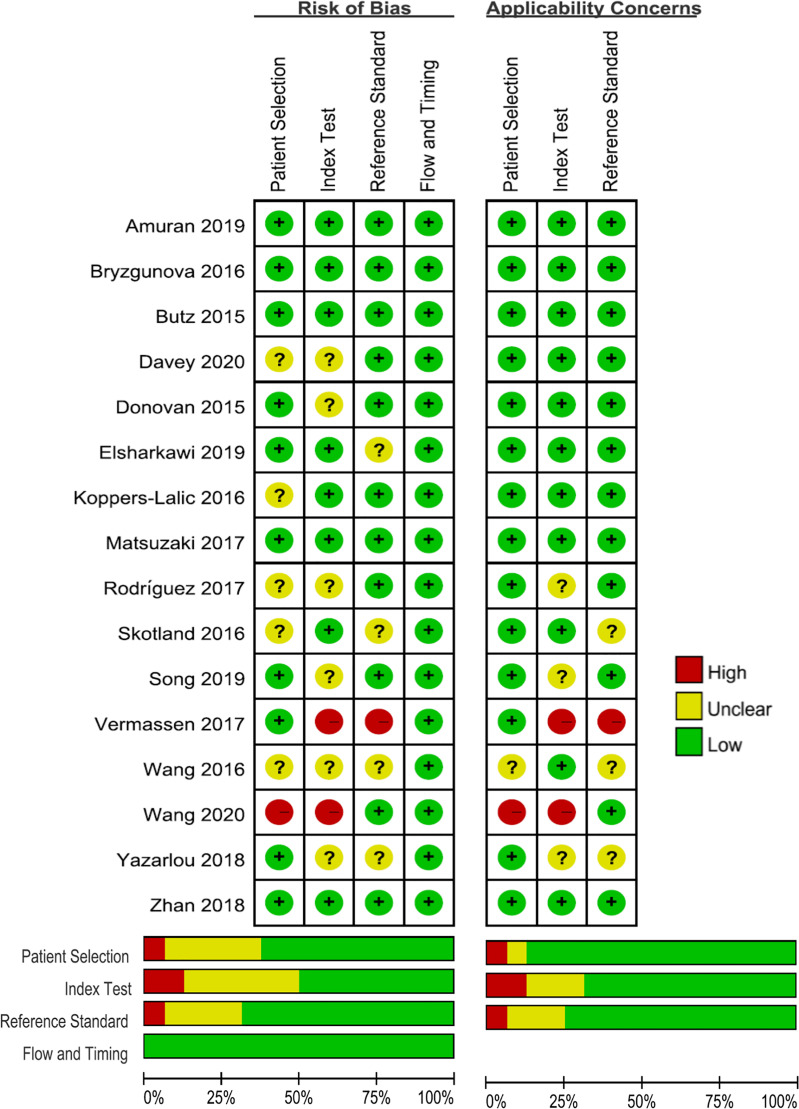
Grouped bar charts show risk of bias and concerns for applicability of 22 included studies using QUADAS-2. QUADAS-2, Quality Assessment of Diagnostic Accuracy Studies-2.

The quality of the selected studies was evaluated in accordance with the QUADAS-2 criteria; the results of these evaluations are shown in **Figure 2**. Five studies were considered to be low-risk with regards to bias and applicability, and the other 11 studies were estimated as suboptimal for unclear risk in several areas, including patient selection, reference standards, and index testing. Deek’s funnel plot was also used to evaluate the publication bias of included studied, and no publication bias was found (P = 0.81) (**Supplementary Figure 1**).

In addition, meta-regression analyses were performed to analyze the heterogeneity with the potential variables, and the type of exosome content (nucleic acid/non-nucleic acid), the type of urological cancer (BCa/PCa/RCa), and proportion of patients with urological cancer (>50%/≤50%) were not significant factors affecting the heterogeneity (P > 0.05, **Supplementary Table 1**).

### Meta Analysis of Diagnostic Value

**Figure 3:**
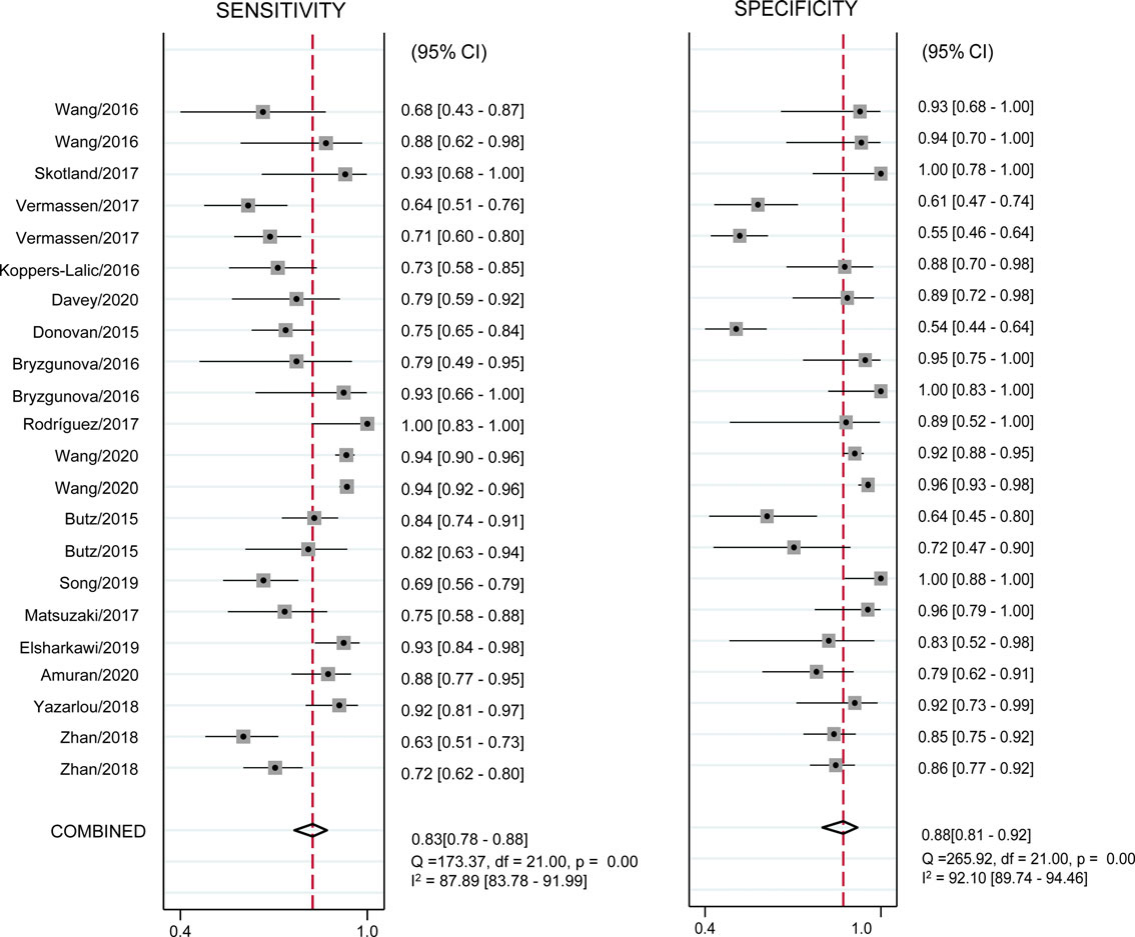
Coupled forest plots of pooled sensitivity and specificity. Numbers are pooled estimates with 95% CI in parentheses. Corresponding heterogeneity statistics are provided at bottom right corners. Horizontal lines indicate 95% CIs. CI, confidence intervals.

**Figure 4:**
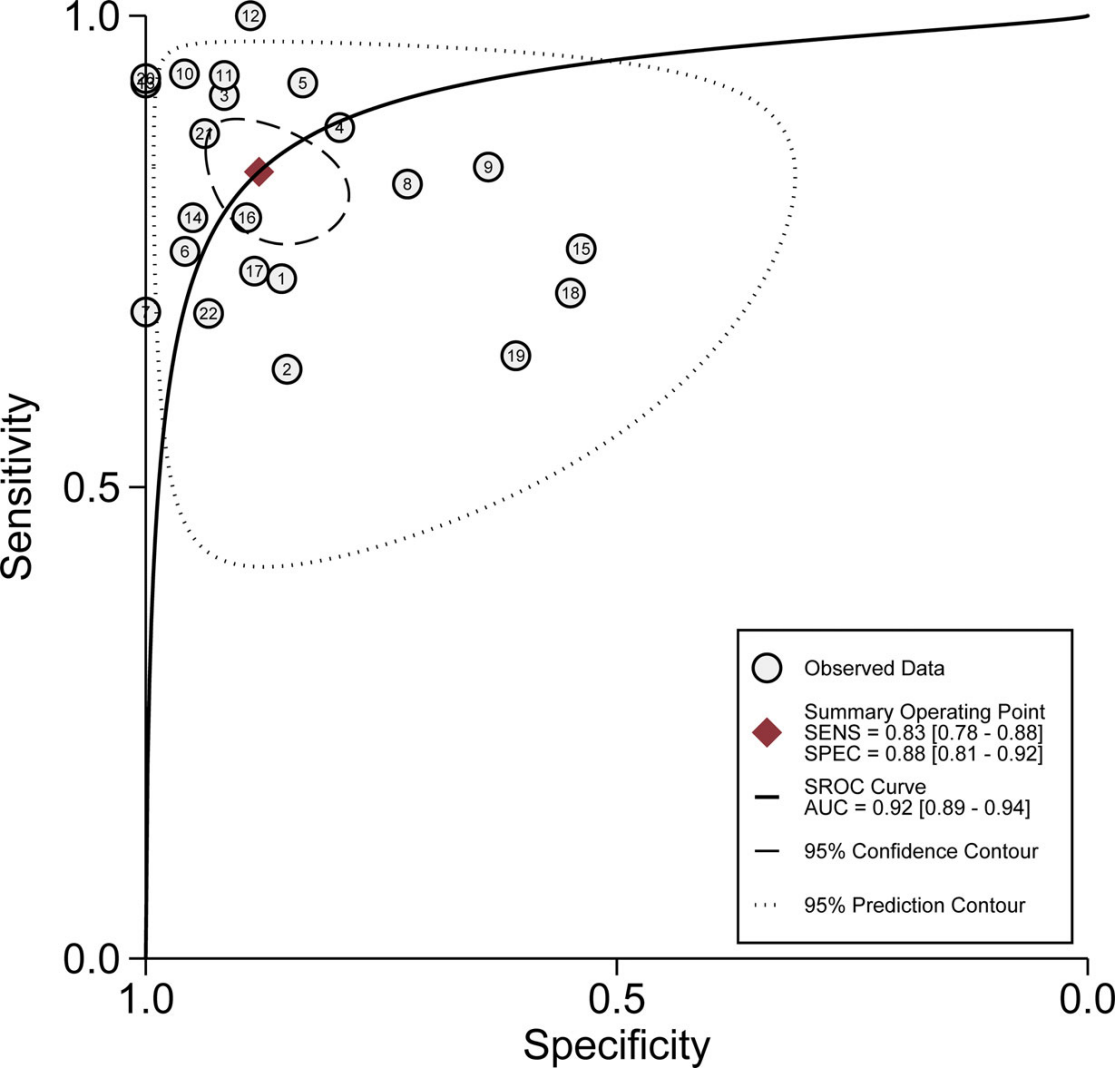
Hierarchical summary receiver operating characteristic curve of the diagnostic performance of urinary exosomes for detecting urological tumor.

All 22 eligible studies were used to evaluate the diagnostic accuracy between urinary exosome expression and urological tumors. As shown in **Figure 3**, the overall diagnostic sensitivity and specificity were 0.83 (95% CI, 0.78–0.88) and 0.88 (95% CI, 0.81–0.92), respectively. Urinary exosome was significantly correlated with sensitivity (P < 0.01, I^2^ = 87.89%) and specificity (P < 0.01, I^2^ = 92.10%) (**Figure 3**). The area under the SROC curve was 0.92 (95% CI: 0.89–0.94) (**Figure 4**). The pooled PLR was 6.94 (95% CI: 4.29–11.22), and the pooled NLR was 0.19 (95% CI: 0.14–0.26) through random effect model (**Supplementary Figure 2**).

### Subgroup Analysis

**Figure 5:**
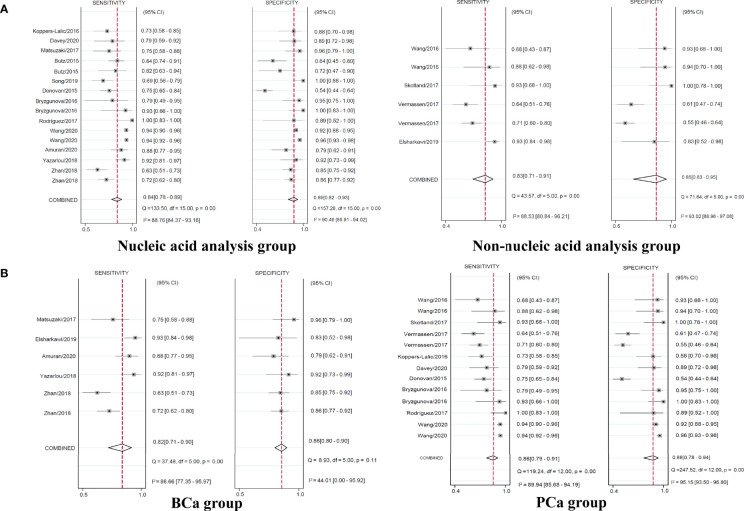
Coupled forest plots of pooled sensitivity and specificity in the subgroup. Numbers are pooled estimates with 95% CI in parentheses. Corresponding heterogeneity statistics are provided at bottom right corners. Horizontal lines indicate 95% CIs. CI, confidence intervals.

When the studies were separately assessed according to the type of exosome content, nucleic acid analysis group of 12 studies yielded pooled sensitivity of 0.84 (95% CI 0.78–0.89) with specificity of 0.89 (95% CI 0.82–0.93), whereas non-nucleic acid analysis group of four studies yielded pooled sensitivity of 0.83 (95% CI 0.71–0.91) with specificity of 0.85 (95% CI 0.63–0.95) (**Figure 5A**).

Regarding the type of urological tumor, the pooled sensitivity of 0.82 (95% CI 0.71–0.90) with specificity of 0.86 (95% CI 0.80–0.90) in five studies of BCa, the pooled sensitivity of 0.86 (95% CI 0.79–0.91) with specificity of 0.88 (95% CI 0.78–0.94) in nine studies of PCa yielded (**Figure 5B**). The pooled sensitivity and specificity of RCa were unable to analyze with only two studies.

## Discussion

RCa, BCa, and PCa are the main types of urological tumors; their morbidity and mortality rates have continued to rise in recent years (26). Although prostate-specific antigen (PSA) testing has been used as biomarker in prostate cancer diagnosis, prostate biopsies are still essential to make a definite diagnosis since PSA level is low, and it also leads to overdiagnosis and overtreatment (27, 28). Most RCas are still found during other abdominal tests (29). Although the targeted therapy and immunotherapy have become the main treatment for advanced RCa, the complete responses is still low, and the biomarker-based strategies are still missing (30). Urological tumors still lack the key targeted markers such as epidermal growth factor receptor (EGFR) for lung cancer and human epidermal growth factor receptor 2 (HER2) for breast cancer.

Urinary cytology was one kind of the main non-invasive diagnostic methods for urothelial cancers (including bladder cancer, renal pelvis cancer, ureteral cancer, and urethral cancer), but its sensitivity was proved deficient (7–17%), and its diagnostic accuracy for low-grade urothelial cancer was relatively low (31). Compared to shedded tumor cells which are harder to capture in urine, exosomes are continually released into the urine from tumor cells. Exosomes can carry antigens from tumor-derived cells, so tumor-related exosomes can be purified by tumor antigen-bound magnetic beads to improve diagnostic specificity. Moreover, the nucleic acid cargo in exosomes may directly reflect the molecular characteristics of urological tumors. In addition, the concentration of exosome-related proteins in the first-morning urination and the second-morning urination were quite similar, and the exosomes remain intact during long-term storage or at -80°C (32), suggesting that urinary exosomes were stable enough to be examined their nucleic acid or non-nucleic acid cargo.

Urine is easy to obtain and has the advantages of convenience, non-invasive, and repeatability. To systematically evaluate the potential of urinary exosomes as non-invasive markers for urological tumors, we established a meta-analysis including 22 studies from 16 articles with 3224 patients and 1360 healthy controls; the results showed an advanced diagnostic accuracy of urinary exosomes with an AUC of 0.92, a sensitivity of 83%, and a specificity of 88%. The overall PLR value of urological exosome was 6.94, suggesting that the probability of having tumor in a people with a positive test was approximately 7-fold higher than negative controls. Several laboratories including ours have reported some over-expressed proteins in tumor tissues, which are valuable in predicting the prognosis of the urological cancer (33–35). Whether these biomarkers can be detected in urinary exosomes and the use of urinary exosomes for monitoring tumor recurrence are worthy of further investigation.

This meta-analysis study suggests the urinary exosomes may serve as non-invasive biomarkers for urological cancer diagnosis. Several limitations of this study need to be discussed. We also reviewed the study of urinary exosomes in other urological tumors (such as ureteral cancer, renal pelvis cancer, epididymal tumor, and testis pellet cancer), but no relevant results were found. Thus, there is still a lack of relevant studies for some urological tumors with low incidences. Because of the large number of included studies reporting positive results, it is impossible to rule out the possibility of selection bias. The potential variables, including the type of exosome content, the type of urological cancer, and proportion of patients with urological cancer were not significant factors affecting the heterogeneity, but whether other factors (such as primers, kits, and quantitative methods) can contribute to bias remains to be evaluated with the enough data.

## Conclusion

Urinary exosomes has great application potential in the noninvasive diagnosis and monitoring of urological tumors. Future evolutions will be necessary to validate whether urinary exosomes may serve as a potential non-invasive marker for early diagnosis and treatment response.

## Data Availability Statement

The original contributions presented in the study are included in the article/**Supplementary Material**. Further inquiries can be directed to the corresponding authors.

## Author Contributions

Two researchers YX and JL independently assessed the eligibility of each potential study by screening the titles and abstracts. Any disagreements between the two researchers were resolved by discussion with two additional researchers HW and ZW. The manuscript was written by YX, YJ, and HX, and AZ, YX, and GL revised. JL, MY, YG, and YH were responsible for the statistical analysis. All authors contributed to the article and approved the submitted version.

## Funding

This work was supported by the National Natural Science Foundation of China (Reference number: 81402117), Natural Science Foundation of Zhejiang Province (Reference number: LY17H160043), and Medical and Health Project of Zhejiang Province (Reference number: 2016KYA038).

## Conflict of Interest

The authors declare that the research was conducted in the absence of any commercial or financial relationships that could be construed as a potential conflict of interest.

## Publisher’s Note

All claims expressed in this article are solely those of the authors and do not necessarily represent those of their affiliated organizations, or those of the publisher, the editors and the reviewers. Any product that may be evaluated in this article, or claim that may be made by its manufacturer, is not guaranteed or endorsed by the publisher.
