# Supplementary figures and images for: Urinary Exosomes Diagnosis of Urological Tumors: A Systematic Review and Meta-Analysis
Source: Front Oncol. 2021 Sep 10;11:734587. doi: 10.3389/fonc.2021.734587 (PMC8462303; doi:10.3389/fonc.2021.734587)

# Deeks' Funnel Plot Asymmetry Test

pvalue = 0.81

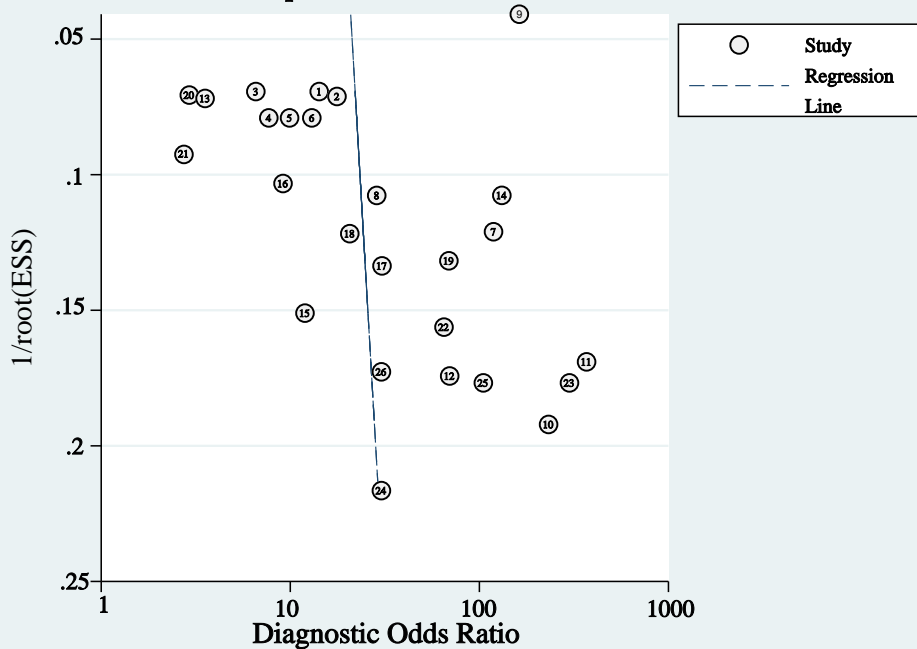

Supplement: Supplementary file 1 [file Image_1.pdf]

## DLR POSITIVE

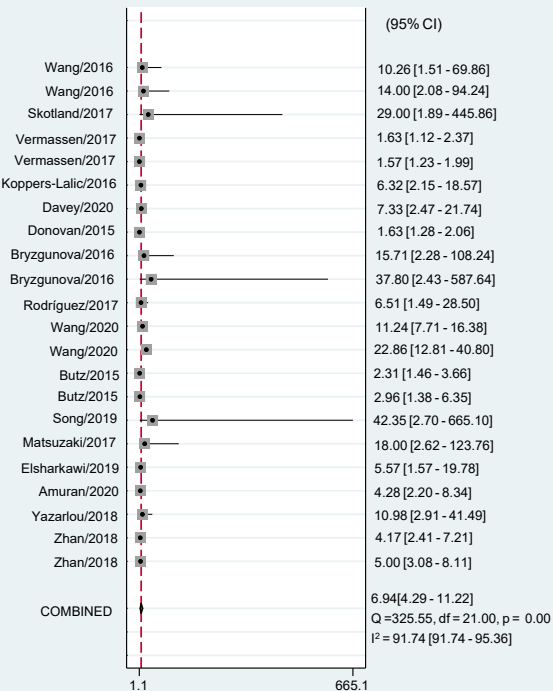

## DLR NEGATIVE

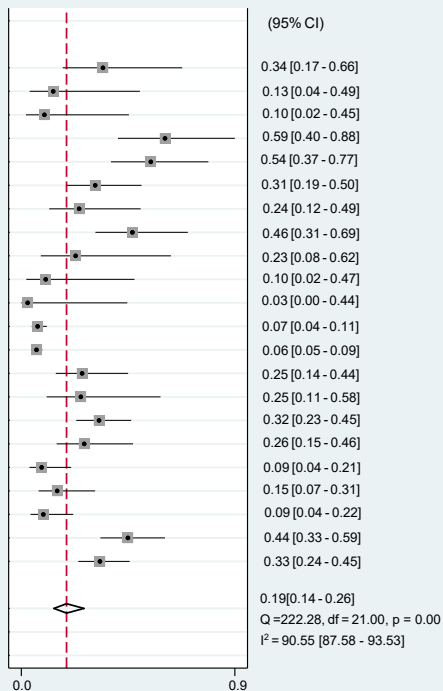

Supplement: Supplementary file 2 [file Image_2.pdf]
