# Supplementary material for: Urinary Exosomes Diagnosis of Urological Tumors: A Systematic Review and Meta-Analysis
Source: Front Oncol. 2021 Sep 10;11:734587. doi: 10.3389/fonc.2021.734587 (PMC8462303; doi:10.3389/fonc.2021.734587)
Supplement: Supplementary file 3 [file Table_1.docx]

**Supplementary Table 1.** Results of meta-regression analysis of urinary exosomes for Detection of Urological Tumor

| Covariates | Subgroup | Meta-analytic Summary Estimates | | |
| --- | --- | --- | --- | --- |
|  |  | Sensitivity  (95% CI) | Specificity  (95% CI) | *p* |
| Proportion of patients with Prostate cancer (%) | >50% | 0.82 (0.75 - 0.88) | 0.86 (0.76 - 0.92) | 0.24 |
|  | ≤50% | 0.83 (0.77 - 0.87) | 0.83 (0.72 - 0.89) |  |
| Type of biomarker | Nucleic acid | 0.90 (0.86 - 0.94) | 0.73 (0.59 - 0.86) | 0.44 |
|  | Non-nucleic acid | 0.89 (0.81 - 0.97) | 0.72 (0.44 - 1.00) |  |
| Type of cancer | BCa | 0.82 (0.77 - 0.86) | 0.79 (0.72 - 0.85) | 0.82 |
|  | PCa | 0.85 (0.76 - 0.90) | 0.88 (0.76 - 0.94) |  |
|  | RCa | - | - |  |

Aberrations: CI = Confidence Intervals, BCa=Bladder Cancer, PCa = Prostate Cancer, RCa = Renal Cancer
